# Multiband Omnidirectional Invisibility Cloak

**DOI:** 10.1002/advs.202401295

**Published:** 2024-05-20

**Authors:** Xiaojun Hu, Yu Luo, Jie Wang, Jingxin Tang, Yuan Gao, Jianhua Ren, Huilong Yu, Jingjing Zhang, Dexin Ye

**Affiliations:** ^1^ Laboratory of Applied Research on Electromagnetics Zhejiang University Hangzhou 310027 China; ^2^ National Key Laboratory of Microwave Photonics Nanjing University of Aeronautics and Astronautics Nanjing 211106 China; ^3^ Laboratory of Antenna Feed System Beijing Institute of Remote Sensing Equipment Beijing 100854 China; ^4^ Institute of Electromagnetic Space State Key Laboratory of Millimeter Waves Southeast University Nanjing 210096 China

**Keywords:** Fabry‐Pérot resonance, multiband, nonmagnetic, omnidirectional invisibility cloak

## Abstract

Transformation optics (TO) provides a powerful tool to manipulate electromagnetic waves, enabling the design of invisibility cloaks, which can render objects invisible. Despite many years of research, however, invisibility cloaks experimentally realized thus far can only operate at a single frequency. The narrow bandwidth significantly restricts the practical applications of invisibility cloaks and other TO devices. Here, a general design strategy is proposed to realize a multiband anisotropic metamaterial characterized by two principal permittivity components, i.e., one infinite and the other spatially gradient. Through a proper transformation and combination of such metamaterials, an omnidirectional invisibility cloak is experimentally implemented, which is impedance‐matched to free space at multiple frequencies. Both far‐field numerical simulations and near‐field experimental mappings confirm that this cloak can successfully suppress scattering from multiple large‐scale objects simultaneously at 5 and 10 GHz. The design strategy and corresponding practical realization bring multiband transformation optical devices one step closer to reality.

## Graphical abstract

Previous invisibility cloaks experimentally realized thus far can only operate at a single frequency. This study reports a multiband omnidirectional invisibility cloak in free space. The cloak uses a nonmagnetic inhomogeneous anisotropic metamaterial working at Fabry–Pérot resonances to replace the transformation‐invariant metamaterial involved in the coordinate transformation, achieving nearly perfect impedance matching and phase restoration in both C and X bands.

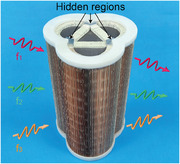

## 1 Introduction

The invisibility cloak, able to hide macroscopic objects in free space, has significant scientific and technological implications. In recent years, the research on invisibility cloaks has developed rapidly^[^ ^1^, ^2^, ^3^, ^4^, ^5^, ^6^, ^7^, ^8^, ^9^, ^10^, ^11^ ^]^ owing to the proposal of transformation optics (TO).^[^ ^12^, ^13^ ^]^ The TO approach originates from the concept that arbitrary coordinate transformations do not alter the form of Maxwell's equation but only change the constitutive parameters and field values. Such a concept provides a powerful tool to control electromagnetic (EM) fields at will.^[^ ^14^, ^15^ ^]^ Despite the significant theoretical developments, however, the constitutive parameters derived from TO are generally inhomogeneous and anisotropic, sometimes even have singular values, and hence are difficult to realize experimentally. To mitigate difficulties in realizing such extreme constitutive parameters, the TO devices are often implemented in a reduced manner (i.e., neglecting the impedance matching and considering only the implementation of the refractive index) at the expense of degrading the device performance.^[^ ^10^, ^16^, ^17^, ^18^, ^19^ ^]^

Recently, a special type of anisotropic material has been introduced, dubbed transformation‐invariant metamaterials (TIMs)^[^ ^20^ ^]^ or optical null media,^[^ ^21^, ^22^, ^23^ ^]^ of which the constitutive parameters are near infinity along the optic axis and near zero along the other two orthogonal directions. TIMs can transfer all incoming waves from the input surface to the output one without introducing phase changes or reflection. Moreover, an arbitrary coordinate transformation applied to TIMs changes only the orientation of the optic axis while retaining the principal values of the permittivity and permeability tensors. These two properties make TIMs an ideal platform to implement full‐parameter TO devices.^[^ ^20^ ^]^ In the practical implementation, TIMs for the transverse magnetic (TM) wave incidence can be realized through either a multilayer of metal/epsilon‐near‐zero metamaterial alternating structures^[^ ^7^, ^10^, ^20^, ^24^ ^]^ or a subwavelength metallic slot array working at the Fabry‐Pérot (FP) resonance frequencies.^[^ ^5^, ^25^, ^26^ ^]^ Nonetheless, all full‐parameter cloaks experimentally implemented thus far only work at a single frequency.^[^ ^2^, ^5^, ^7^ ^]^ Although the carpet cloak has a broad operating bandwidth,^[^ ^8^, ^27^ ^]^ it only works for a finite range of illumination angles and generally requires the object to sit on a conducting background. Other theoretical studies have explored the possibilities of extending the bandwidth of omnidirectional cloaks,^[^ ^28^, ^29^, ^30^, ^31^ ^]^ but corresponding experimental realizations are yet to be reported. Up to today, it is still challenging to realize a multiband cloak with ideal invisibility performance in the air.

Here, we present the practical realization of a full‐parameter omnidirectional cloak that can significantly suppress the scattering from a large‐scale object at multiple frequencies. Our design uses a special type of multiband anisotropic metamaterials consisting of subwavelength metallic slots filled with dielectrics with gradient thicknesses. Such a metamaterial has an anisotropic spatially inhomogeneous permittivity tensor. Through a judicious design, it fulfills the requirements of omnidirectional impedance matching to free space at multiple frequencies. As an experimental proof, we fabricate a cloak sample and map out the scattered magnetic field distributions through direct field measurements. Both numerical simulations and experimental measurements confirm excellent cloaking performance at 5 GHz and 10 GHz. Our design strategy and practical implementation can be easily extended to higher frequency bands, such as terahertz frequencies, showing great potential in multiwavelength systems.

## 2 Results

**Figure 1:**
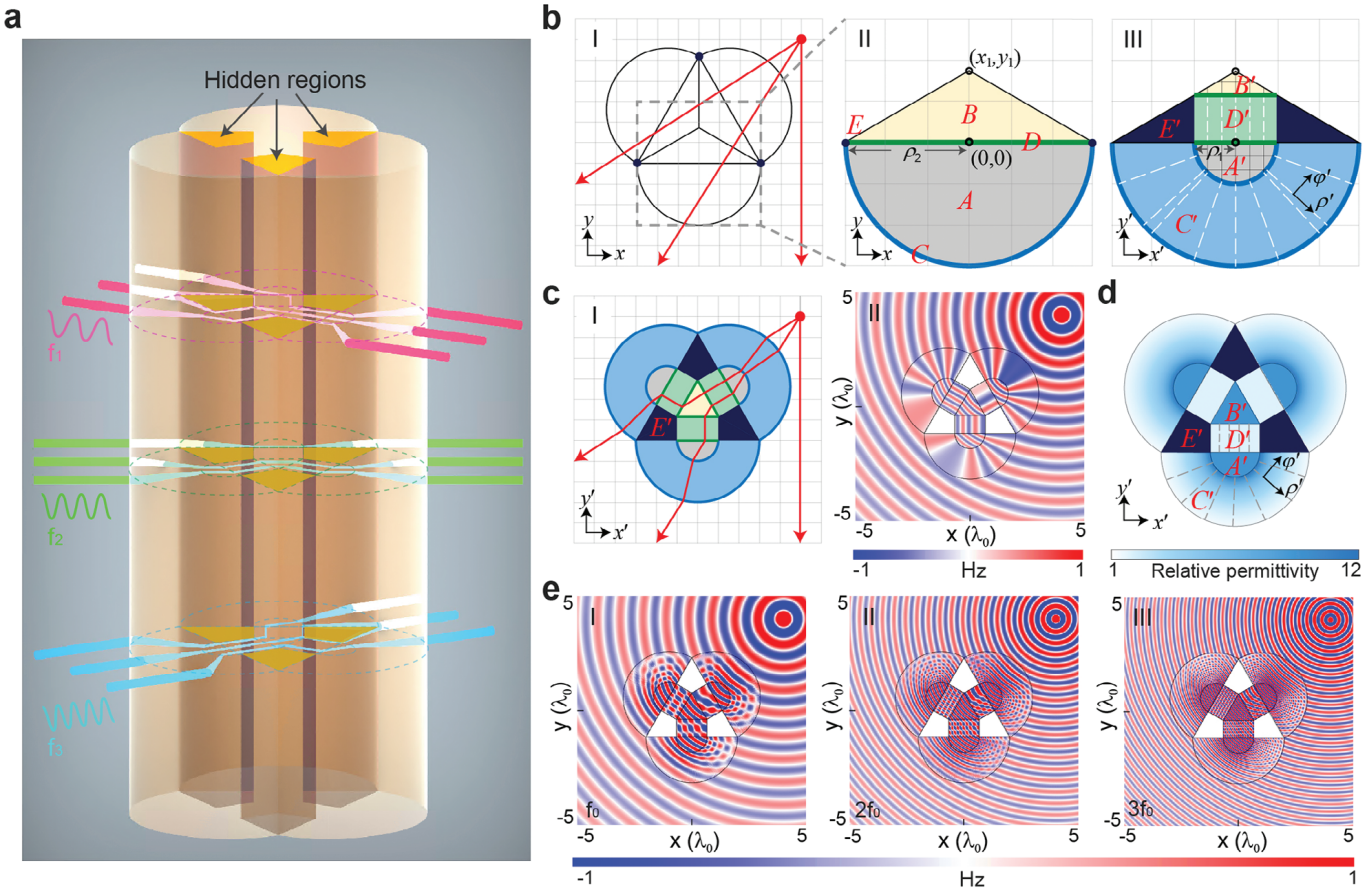
Multiband omnidirectional cloak design. a) Schematic view. b) Coordinate transformation. Panels I and II show the initial space, and its details with enlarged scale, respectively. Panel III shows the correspondence between the initial space and the physical space. c) The ray tracing (I) with light from a point source passing through the magnetic cloak composed of TIMs and the magnetic medium defined in Equations (5)–(7), and the corresponding simulated magnetic field distribution (II). d) Permittivity profile of the nonmagnetic cloak composed of FP media and the normal dielectric defined in Equations (8)–(12). e) Simulated magnetic field distributions around the nonmagnetic cloak at *f*_0_ (I), 2*f*_0_ (II) and 3*f*_0_ (III).

**Figure 2:**
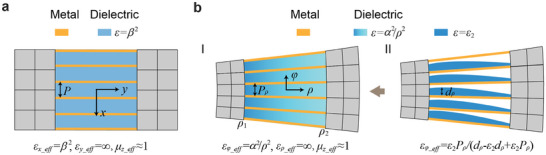
Physical implementation of the desired FP media. a) The homogeneous planar FP medium in the Cartesian coordinate using metallic slot arrays filled with the dielectric slabs (*ε* = *β* ^2^). The width of each slot is denoted as *P* (<< *λ*_0_), and the thickness of each metallic sheet is extremely small (<<*P*). b) The inhomogeneous annulus FP medium in the cylinder coordinate using metallic slot arrays fully filled with (I) an inhomogeneous dielectric (*ε* = *α* ^2^/*ρ* ^2^), and partially filled with (II) homogeneous dielectric slabs (*ε* = *ε*_2_) with gradient thicknesses *d_ρ_*. The formulas at the bottoms show the effective permittivities according to the Maxwell Garnett mixing rule.^[^ ^32^ ^]^

**Figure 3:**
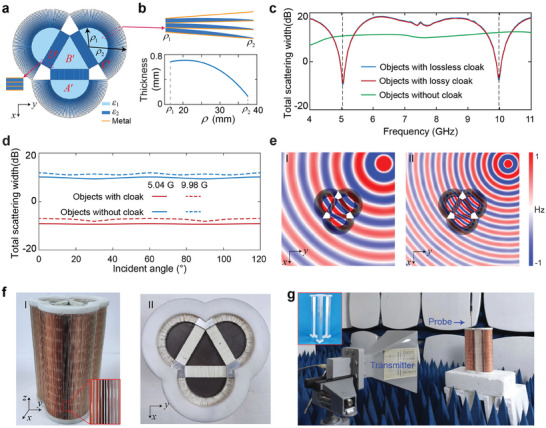
Actual design of the multiband cloak. a) The schematic of the cloak comprised only metallic sheets and two dielectrics, where *ε*_1_ = 3.88, *ε*_2_ = 6.15, *ρ*_1_ = 21.58 mm, and *ρ*_2_ = 42.53 mm. Three identical white quadrilaterals denote the cloak regions. b) Details of the inhomogeneous annulus FP structure and the gradient thickness of the dielectric slab varying with the radius *ρ*. c) Simulated normalized total scattering widths of the actual lossy cloak (red line) and the lossless one (blue line) varying with the frequency. The green line denotes the result for the bare object without cloak. d) Simulated normalized total scattering widths of the actual lossy cloak varying with the incident angle at 5.04 and 9.98 GHz (red lines), with the blue lines denoting the result for the bare object without cloak. e) Simulated magnetic field distributions while placing a point source near the actual lossy cloak at 5.04 GHz (I) and 9.98 GHz (II). f) Photographs of the fabricated cloak. g) Experimental setup for measuring the magnetic field distributions. The inset shows the fabricated three quadrangular metals for comparison.

**Figure** **1**a shows the schematic view of the proposed cloak. It renders the multiwavelength EM waves to travel around three hidden regions without disturbing their phases and amplitudes, regardless of the incident angles. Figure 1b illustrates the transformation principle, of which panel I shows light rays (red arrows) from a point source propagating in free space. The cloak is obtained through a two‐step transformation applied to the region comprised of 3 triangles and 3 semicircles (see dark lines in panel I). Since the region to be transformed has a C3 symmetry, we only consider the transformation applied to the triangle and semicircle at the bottom. In the first step, the large semicircle with a radius *ρ*_2_ (i.e., gray region *A* in panel II) is compressed into a small semicircle with a radius *ρ*_1_ (i.e., gray region *A*′ in panel III), while an infinitely thin annulus region with inner and outer radii both equal to *ρ*_2_ (i.e., the blue outline *C* in panel II) is stretched into a finite annulus region with an inner radius *ρ*_1_ and an outer radius *ρ*_2_ (the blue region *C*′ in panel III). In the second step, the large triangle (i.e., yellow region *B* in panel II) is compressed into a small triangle (i.e., yellow region *B*′ in panel III), while the green straight line denoted by *D* in panel II is first compressed along the horizontal and then stretched along the vertical to form the green rectangular region *D*′ in panel III. By combining the 3 triangular and 3 semicircular regions together, such a two‐step transformation maps the 3 deep blue points *E* at the corners of the large equilateral triangles in panel I of Figure 1b to 3 deep blue regions *E*′ in panel I of Figure 1c. Panel I of Figure 1c also illustrates how light rays are smoothly guided without any reflection or deflection around the 3 deep blue regions, inside which arbitrary objects can be effectively concealed. This two‐step transformation can also be expressed by the following equations:

(1) $$\rho^{\prime }=\frac{\rho_{1}}{\rho_{2}}\rho ,\varphi^{\prime }=\varphi ,z^{\prime }=z\;\mathrm{for}\;A\to A^{\prime }$$

(2) $$\rho^{\prime }=\rho_{1}{\left(\frac{\rho_{2}}{\rho_{1}}\right)}^{\frac{\rho -\rho_{2}}{\Delta }},\varphi^{\prime }=\varphi ,z^{\prime }=z\;\mathrm{for}\;C\to C^{\prime }$$

(3) $$x^{\prime }=x_{1}+\frac{\rho_{1}}{\rho_{2}}\left(x-x_{1}\right),y^{\prime }=y_{1}+\frac{\rho_{1}}{\rho_{2}}\left(y-y_{1}\right),z^{\prime }=z\;\mathrm{for}\;B\to B^{\prime }$$

(4) $$x^{\prime }=\frac{\rho_{1}}{\rho_{2}}x,y^{\prime }=\left(\rho_{2}-\rho_{1}\right)\frac{y_{1}y}{\rho_{2}\Delta },z^{\prime }=z\;\mathrm{for}\;D\to D^{\prime }$$

where Δ is infinitesimal; (*x*, *y*, *z*) and (*ρ*, *φ, z*) denote the Cartesian and cylinder coordinates, respectively; unprimed and primed coordinate systems correspond to the initial and physical spaces.

For TM‐polarized waves with a magnetic field perpendicular to the *x–y* plane, the constitutive parameters of the transformed materials in the physical space can be obtained as:

(5) $$\varepsilon_{x^{\prime }}=\varepsilon_{y^{\prime }}=1,\mu_{z^{\prime }}=\frac{\rho_{2}^{2}}{\rho_{1}^{2}}\;\mathrm{for}\;A^{\prime }\;\mathrm{and}\;B^{\prime }$$

(6) $$\begin{array}{ccc} \varepsilon_{\rho^{\prime }} & = & \frac{\rho_{2}\ln\left(\rho_{2}/\rho_{1}\right)}{\Delta }\to \infty ,\varepsilon_{\varphi^{\prime }}=\frac{\Delta }{\rho_{2}\ln\left(\rho_{2}/\rho_{1}\right)}\to 0,\mu_{z^{\prime }}\\ & = & \frac{\rho_{2}\Delta }{{\rho^{\prime }}^{2}\ln\left(\rho_{2}/\rho_{1}\right)}\to 0\;\mathrm{for}\;C^{\prime } \end{array}$$

(7) $$\begin{array}{ccc} \varepsilon_{x^{\prime }} & = & \frac{\rho_{1}\Delta }{\left(\rho_{2}-\rho_{1}\right)y_{1}}\to 0,\varepsilon_{y^{\prime }}=\frac{\left(\rho_{2}-\rho_{1}\right)y_{1}}{\rho_{1}\Delta }\to \infty ,\mu_{z^{\prime }}\\ & = & \frac{{\rho_{2}}^{2}\Delta }{\left(\rho_{2}-\rho_{1}\right)\rho_{1}y_{1}}\to 0\;\mathrm{for}\;D^{\prime } \end{array}$$

The constitutive parameters for regions *C*′ and *D*′ are infinite along the optic axis (i.e., *ρ*′ and *y*′ directions, denoted by the white dashed lines in panel III of Figure 1b) and near zero along the other two orthogonal directions. Such a material, called TIM, can guide light along the optic axis without introducing any reflection or phase distortion. A cloak is obtained by properly gluing three pieces of such materials together. Using the commercial software COMSOL Multiphysics, we perform a full‐wave simulation for this cloak. The simulated magnetic field distributions in panel II of Figure 1c show that the EM waves incident from a point source onto the cloak are smoothly guided around the 3 deep blue regions and then return to their original path, as if it has just passed through vacuum. Although this result confirms the omnidirectional cloaking effect, the extreme constitutive parameters encountered are still quite difficult, if not impossible, to implement even at a single frequency. First, TIMs in regions *C*′ and *D*′ acquire anisotropic extreme permittivities and zero permeability. Second, regions *A*′ and *B*′ acquire a magnetic material, whose permeability is larger than 1 and permittivity is equal to 1.

To ease the practical implementation of the cloak designed above, we search for a way to eliminate the magnetic responses by noting that the constitutive parameters given by Equations (5)–(7) are equivalent to the following:

(8) $$\varepsilon_{x^{\prime }}=\varepsilon_{y^{\prime }}=\frac{\rho_{2}^{2}}{\rho_{1}^{2}},\mu_{z^{\prime }}=1\;\mathrm{for}\;A^{\prime }\;\mathrm{and}\;B^{\prime }$$

(9) $$\varepsilon_{\rho^{\prime }}\to \infty ,\varepsilon_{\varphi^{\prime }}=\frac{\alpha^{2}}{{\rho^{\prime }}^{2}},\mu_{z^{\prime }}=1\;\mathrm{for}\;C^{\prime }$$

(10) $$\varepsilon_{x^{\prime }}=\beta^{2},\varepsilon_{y^{\prime }}\to \infty ,\mu_{z^{\prime }}=1\;\mathrm{for}\;D^{\prime }$$

where *α* and *β* are two undetermined constants. Now, we pause to analyze the similarities between Equations (5)–(7) and Equations (8)–(10). First, the media characterized by Equations (9) and (10) have extremely anisotropic permittivity tensors, indicating that EM waves can only propagate along the direction of the optic axis, i.e., the *ρ*′ and *y*′ directions. Second, the dielectric defined by Equation (8) has the same refractive index *n* = *ρ*_2_/*ρ*_1_ as the magnetic material defined by Equation (5), and hence, light rays going into or out of these two media will be refracted at the same angle. In other words, the media given by Equations (8)–(10) and Equations (5)–(7) can guide light in a similar manner. Hence, Equations (8)–(10) provide an alternative way to realize omnidirectional invisibility as long as the reflection at the boundary of each region can be eliminated. This can be done by properly setting the two constants *α* and *β*, such that EM waves propagating in regions *C*′ and *D*′ satisfy the FP condition (See Sections SI and SII, Supporting Information for detailed numerical proofs). This condition requires phase accumulation along the optic axis of the medium to be integer times of 2π, namely,

(11) $$\int_{\rho_{1}}^{\rho_{2}}\sqrt{\varepsilon_{\varphi^{\prime }}}d\rho^{\prime }=\alpha \ln\left(\frac{\rho_{2}}{\rho_{1}}\right)=N_{1}\lambda_{0}\Rightarrow \alpha =\frac{N_{1}\lambda_{0}}{\ln\left(\rho_{2}/\rho_{1}\right)}$$

(12) $$\sqrt{\varepsilon_{x^{\prime }}}\times \frac{\rho_{2}-\rho_{1}}{\sqrt{3}}=N_{2}\lambda_{0}\Rightarrow \beta =\frac{\sqrt{3}N_{2}\lambda_{0}}{\rho_{2}-\rho_{1}}$$

Here, *λ*_0_ denotes the operating wavelength in free space; *N*_1_ and *N*_2_ can be any arbitrary nonzero integer or half‐integer. Note that EM waves coming from any direction will pass through regions *C*′ and *D*′ twice, and consequently, the total phase accumulation across the whole cloak is always integer times of 2π even if *N*_1_ and *N*_2_ are half integer. As an example, we design a cloak by setting *ρ*_1_ = 2*λ*_0_/3, *ρ*_2_ = 2*λ*_0_, *N*_1_ = 2.5, and *N*_2_ = 1. Figure 1d shows the permittivity profile of this cloak (note that the contour plots in regions *C*′ and *D*′ denote the permittivity component perpendicular to the optic axis). Apparently, the involved permittivities are larger than 1. In this way, an omnidirectional cloak is obtained without involving any magnetic materials.

More importantly, such a cloak inherently operates at a series of frequencies, satisfying Equations (8)–(12). To illustrate this point, Figure 1e plots the simulated field distributions around this cloak at the first three resonance frequencies (panel I for *f*_0_, II for 2*f*_0_, and III for 3*f*_0_) by placing a point source with a *z*‐polarized magnetic field close to the cloak. No noticeable wave distortion is observed at any of these frequencies, demonstrating the multiband invisibility behavior.

Physical implementation of the designed nonmagnetic cloak relies on the realization of the two extremely anisotropic media in regions *C*′ and *D*′. The material in region *D*′ is homogeneous and hence can be easily implemented with a subwavelength metallic slot array filled with a normal dielectric,^[^ ^5^ ^]^ as shown in **Figure** **2**a. The width of the slots is denoted as *P* (<< *λ*_0_), the thickness of each metallic sheet is much smaller than *P*, and the permittivity of the filling dielectric is *ε* = *β* ^2^. When *β* is given by Equation (12), the effective constitutive parameters of the metallic slot array satisfy Equation (10).^[^ ^26^ ^]^ Since the material in region *C*′ acquires an inhomogeneous *ε_φ_*_′_, its implementation requires filling the metallic slot array with an inhomogeneous dielectric (*ε_φ_*_′_ = *α* ^2^/*ρ* ^2^), as shown in panel I of Figure 2b. However, such an inhomogeneous dielectric is generally not available in nature. To find a simple solution to this problem, we apply the Maxwell Garnett mixing rule^[^ ^32^ ^]^ and attain the anisotropic inhomogeneous metamaterial in region *C*′ by filling each metallic slot with a homogeneous high‐permittivity dielectric (*ε* = *ε*_2_) material with a non‐uniform thickness, as shown in panel II of Figure 2b. Such a metamaterial has constant permittivity/permeability components along the  *ρ* and *z* directions, i.e., *ε_ρ_eff_* = ∞ and *μ_z_eff_* ≈ 1, and a thickness‐dependent permittivity component along the *φ* direction, i.e., $\varepsilon_{\varphi \_eff}=\frac{\varepsilon_{2}P_{\rho }}{d_{\rho }-\varepsilon_{2}d_{\rho }+\varepsilon_{2}P_{\rho }}$,where *P_ρ_* and *d_ρ_* denote the slot period and the dielectric thickness, respectively. Assuming that the angle between neighboring metallic sheets is *θ_del_* (hence, *P_ρ_* = *ρθ_del_*) and set $d_{\rho }=\frac{\varepsilon_{2}\rho \theta_{del}\left(\alpha^{2}-\rho^{2}\right)}{\left(\varepsilon_{2}-1\right)\alpha^{2}}$, the desired *ε_φ_*___ *_eff_* = *α* ^2^/*ρ* ^2^ is achieved as long as $\rho_{2}<\alpha <\sqrt{\varepsilon_{2}}\rho_{1}$. It should be noted that the effective permittivity of the FP structure composed of homogeneous dielectric slabs with gradient thicknesses in region *C*′ is in principle anisotropic, i.e., its three components *ε_ρ_*___ *_eff_*, *ε_φ_*___ *_eff_* and *ε_z_*___ *_eff_* are different from each other. However, under the TM wave incidence, *ε_z_*___ *_eff_* is not involved and thus can be ignored.

Based on the approach given in Figure 2, we design an actual cloak composed of copper sheets and two homogeneous dielectrics, as shown in **Figure** **3**a. Here, *ρ*_1_ = 21.58 mm, *ρ*_2_ = 42.53 mm. The pale blue regions (regions *A*′and *B*′) denote the F4B dielectric (*ε*_1_ = 3.88, with a loss tangent of 0.0015), the deep blue regions denote the Rogers *RO*4360G2 dielectric (*ε*_2_ = 6.15, with a loss tangent of 0.0038), and the orange lines denote the thin copper sheets with a thickness of 0.035 mm. In the simulation, the permittivity of copper is described by the Drude model, i.e., *ε*_*r*_ = 1 – *ω*_*p*_^2^/(*ω* ^2^ + *iω* *γ*), where the plasma frequency *ω*_*p*_ and the damping frequency *γ* are 1.34 × 10^16^ and 1.45 × 10^14^ rad s^−1^, respectively.^[^ ^33^ ^]^ In such a case, both the material losses and the frequency dispersion of metals are considered. The planar structure in region *D*′ is constructed by closely stacking 54 copper‐dielectric bilayers with a periodicity of 0.8 mm, and its details are schematically shown in the inset of Figure 3a. The annulus FP structure (region *C*′) is composed of 90 copper‐dielectric bilayers (*θ_del_* = π/90) with a periodicity of 0.753 mm at *ρ* = *ρ*_1_, whose details are schematically shown in the top panel of Figure 3b. The bottom panel of Figure 3b shows the thickness *d_ρ_* of the Rogers *RO*4360G2 slab dependent on *ρ*, with *d_ρ_* = 4.17 × 10^−2^ *ρ* – 2.13 × 10^−5^ *ρ* ^3^ (see Section SIII, Supporting Information for more details). Such parameters ensure that all constitutive parameters satisfy Equations (8)–(12) with *N*_1_ = *N*_2_ = 0.5 at 5 GHz. Figure 3c shows the simulated total scattering width (normalized by the outer radius (*ρ*_2_) of the annulus FP structure) of this actual lossy cloak varying with the frequency (red line), calculated according to *σ_norm_*_._ = $\lim_{\rho \to \infty }\int_{0}^{2\pi }2\pi \rho \cdot |H_{s}^{2}\left|/\right|H_{i}^{2}|\cdot d\varphi /\rho_{2}$. Here, *H_s_* and *H_i_* denote the scattered and incident magnetic field intensities, respectively. For comparison, we also show the result of the bare object without cloak (green line), i.e., three small quadrangular metals. Meanwhile, to show the impacts of material losses and the frequency dispersion of copper, the total scattering width of the lossless cloak composed of PEC sheets and lossless dielectrics is also given (blue line). As we expect, multiband cloaking performances (the scattering suppressions ≈20 dB at two optimal operating frequencies, i.e., 5.04 and 9.98 GHz) are observed over a wide range of illumination angles for both the lossless cloak and the lossy one. These results clearly demonstrate that the influences of material losses, frequency dispersion, and plasmonic effects of the copper on the cloaking performance are nearly negligible. For detailed illustration, we also simulate the energy dissipations on dielectrics and copper sheets, which are ≈5.4% and 8.5% at 5.04 and 9.98 GHz, respectively (see Figure S3d, Section SIV, Supporting Information). Moreover, to show its omnidirectional cloaking performance, we plot the total scattering widths of this lossy cloak varying with the incident angle *θ* at two frequencies in Figure 3d, showing the expected multiband omnidirectional cloaking performance. Here, *θ* refers to the angle between the incident direction and the *x* axis, and it ranges only from 0° to 120° due to the C3 symmetry of the designed cloak. To further verify the small impact of material losses, we also show the simulated total scattering widths of the lossless cloak (composed of PEC sheets and lossless dielectrics) and the lossy one (composed of copper sheets and lossy dielectrics) in Section SIV (Supporting Information). Finally, Figure 3e shows the simulated magnetic field distributions at 5.04 GHz (I) and 9.98 GHz (II) when a point source with a *z*‐polarized magnetic field is placed near this lossy cloak. The cylindrical wave bypasses three white quadrangular regions (cloak regions), inducing only weak forward scattering. This scattering results from the fact that the copper sheets are not thin enough, such that the effective permeability of regions *C*′ and *D*′ are not strictly equal to 1.

To experimentally verify the invisibility performance of the designed cloak, we fabricate a cloak sample and map out the near‐field distributions in the anechoic chamber. The cloak sample is depicted in Figure 3f, whose geometric parameters are the same as those in the full‐wave simulations shown in Figure 3a,b. Figure 3g shows the experimental setup for the near‐field scanning. Especially, as the inset shows, we fabricate a sample composed of three quadrangular metals (Aluminum) with the same shape and size as the cloak regions for comparison. Details on the sample fabrications and measurement setups can be found in the Experimental Section.

**Figure 4:**
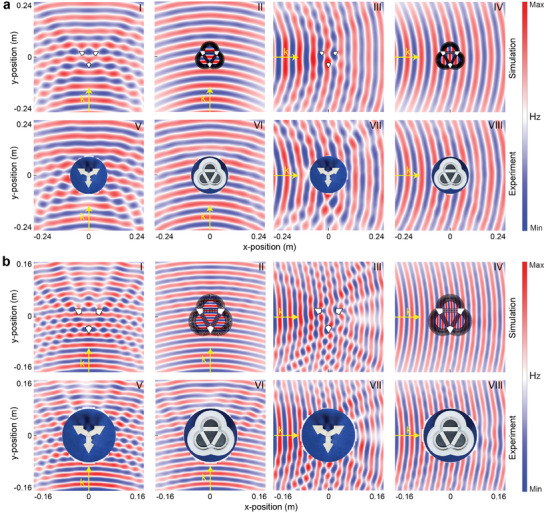
Simulated and measured magnetic field distributions around the designed sample. a) Results at 5 GHz. Panels I–IV show the simulated magnetic field distributions around three identical quadrangular metals without (I, III) and with the designed cloak (II, IV), under different wave incidences. Panels V‐VIII show the corresponding measured results. The incident direction is along the *y* axis for panels I and II, and along the *x* axis for panels III and IV. b) Corresponding results at 10 GHz.

**Figure** **4** shows the full‐wave simulated results (panels I‐IV) and the measured ones (panels V–VIII), where Figure 4a,b show the results at 5 and 10 GHz. Panels I and III show the simulated magnetic field distributions around the quadrangular metals without the cloak when the incident EM wave travels along the *y* and *x* axes, respectively. Panels V and VII show the corresponding measured results. Panels II and IV show the simulated field distributions around the cloak under different wave incidences, while panels VI and VII show the corresponding measured ones. As we see, the scatterings from the quadrangular metals are always strong in both the simulations and the experiments at two frequencies. The standing wave effects agree well with each other, proving the feasibility of the experimental setup. Meanwhile, the simulated and measured results of the cloak at two frequencies are also in good agreement with each other, simultaneously presenting the successful scattering suppression in all directions. For a detailed comparison of the scattered fields, please refer to Figure S5, Section SV (Supporting Information).

It should be noted that the cloaking performance is inevitably deteriorated by fabrication imperfections, especially at 10 GHz. The main reason may be the machining errors in the gradient thickness of the dielectric slabs, which will make the phase accumulations along the optic path deviate from the integer times of 2π. This degradation manifests as improper forward field reconstructions, which is more evident at the higher frequency. Moreover, the arrangement periodicity of the metallic slots may not be small enough. Thus, this invisibility performance might be further improved by decreasing the periodicity of the metallic slots to make the effective metamaterials more uniform. Certainly, it will require more precise machining processing.

## 3 Conclusion

In conclusion, we experimentally demonstrated a multiband omnidirectional cloak for the TM wave incidence. Although fabrication imperfections inevitably degrade the measured results, theoretical analysis, full‐wave simulations, and experiments still agree well with each other. The key to our design is using FP media and normal dielectrics to replace the TIMs and magnetic media involved in the TO‐based cloak, respectively. In particular, the introduction of an inhomogeneous FP medium ensures a nearly perfect impedance matching while keeping the transmitted wave phase undistorted. In principle, this TO‐based cloak has no size and shape restrictions to the cloaked object, so long as the cloak shell is enlarged as the cloaked object grows. This is because the FP media always match the free space at FP resonance frequencies, regardless of their electrical thickness. Moreover, the nonmagnetic constitutive parameters significantly simplify the physical implementation without involving resonant metamaterials with particular constitutive parameters, which only relies on metallic sheets and homogeneous dielectrics. It ensures that the losses of dielectrics and metals have a small impact on the cloaking performance.

Finally, although the experimental measurements are only conducted at 5 and 10 GHz, the designed cloak theoretically covers triple, quadruple, or even more frequency bands (at FP resonance frequencies, such as 15 GHz, 20 GHz, etc.). Considering that the proposed approach only requires a good conductor and some metals such as silver and gold, still behave as good conductors at higher frequencies, this method can be extended to terahertz or even near‐infrared regimes. As an example, we provide a multiband cloak design operating at terahertz frequencies by full‐wave simulations, please refer to Section SVI (Supporting Information) for more details. Our work paves a new route to realize multiband TO devices and shows great application prospects in areas of stealth technology and electromagnetic compatibility, especially demanding omnidirectional incidence and multiple operating frequency bands.

## 4 Experimental Section

### Fabrication Methods

In assembling the cloak, a Nylon base was first fabricated using 3D printer technology, with specifically distributed grooves on the top surface to hold the standing copper‐dielectric composite sheets. Three annulus FP structures were composed of 270 identical composite sheets (20.96 mm in length, 200 mm in height). These sheets were inhomogeneous in thickness, and their thickness function is shown in Figure 3b, processed by a mechanical engraving machine. Three planar FP structures consisted of 156 identical composite sheets (12.10 mm in length, 200 mm in height, and 0.81 mm in thickness). The copper coatings of all composite sheets were 0.035 mm thick, covering only one side of the dielectrics. In the semicircle and triangle regions (regions *A*′ and *B*′ shown in Figure 3a), F4B dielectric sheets with the corresponding shapes (5 mm in thickness, 39 layers in all) were stacked closely layer‐by‐layer. Finally, all the previous components, including three quadrangular Aluminum columns, were hooped with another 3D‐printed Nylon coverer, as shown in Figure 3e.

### Measurement Setup

In the experimental measurements (Figure 4a), a C‐band standard horn antenna, located 60 cm away from the test sample, was used as the transmitter to radiate EM waves with a *z*‐polarized magnetic field. A homemade loop antenna with a radius of 5 mm was used as the receiver to detect the magnitude and phase of the magnetic field point‐to‐point in the *x*‐*y* plane, which was controlled by a mechanical arm. The field scanning area was 480 × 480 mm^2^ with a spatial resolution of 8 × 8 mm^2^. In the measurements for the results shown in Figure 4b, an X‐band standard horn antenna was used as the transmitter, and a 2‐mm‐radius homemade loop antenna was used as the probe. The field scanning area was 320 × 320 mm^2^ with a spatial resolution of 4 × 4 mm^2^.

## Conflict of Interest

The authors declare no conflict of interest.

## Supporting information

Supporting Information

## Data Availability

The data that support the findings of this study are available from the corresponding author upon reasonable request.
