# Supplementary material for: Multiband Omnidirectional Invisibility Cloak
Source: Adv Sci (Weinh). 2024 May 20;11(28):2401295. doi: 10.1002/advs.202401295 (PMC11267388; doi:10.1002/advs.202401295)
Supplement: Supplementary file 1 — Supporting Information [file ADVS-11-2401295-s001.docx]

Supporting Information

Multiband omnidirectional invisibility cloak

Xiaojun Hu, Yu Luo,* Jie Wang, Jingxin Tang, Yuan Gao, Jianhua Ren, Huilong Yu, Jingjing Zhang,* and Dexin Ye*

**I. Fabry-Pérot (FP) medium in the Cartesian coordinate**

In this section, we will prove analytically that a planar homogeneous FP medium in the Cartesian coordinate is fully equivalent to a TIM in arbitrary isotropic backgrounds.

**Figure S1**a shows that an FP medium slab (with a thickness of *L*) is placed in an isotropic background (with the relative permittivity *ε*_1_ and the relative permeability *μ*_1_), whose optic axis is along the *y* direction. A *z*-polarized TM wave is impinging on this FP slab with the incident angle of *θ*, and the involved three constitutive parameters of the FP slab are *ε_x_* = *ε*_2_, *ε_y_* = ∞, *μ_z_* = 1. Then, we can easily derive the reflection (*R*) and transmission coefficients (*T*) as:

$R=\frac{(k_{2y}^{2}-\varepsilon_{2}^{2}k_{1y}^{2})(e^{-2ik_{2y}L}-1)}{(k_{2y}+\varepsilon_{2}k_{1y})^{2}-(k_{2y}-\varepsilon_{2}k_{1y})^{2}e^{-2ik_{2y}L}}$, (S1)

$T=\frac{4\varepsilon_{2}k_{1y}k_{2y}e^{-ik_{2y}L}}{(k_{2y}+\varepsilon_{2}k_{1y})^{2}-(k_{2y}-\varepsilon_{2}k_{1y})^{2}e^{-2ik_{2y}L}}$, (S2)

where $k_{1y}=k_{0}\sqrt{\varepsilon_{1}\mu_{1}}\cos\theta$ and $k_{2y}=k_{0}\sqrt{\varepsilon_{2}}$represent the wave vectors along the *y* direction in the background and the FP slab, respectively. According to Equation (S1) and (S2), *R* will be 0, and *T* will be 1, regardless of the incident angle *θ*, as long as $e^{-ik_{2y}L}=1$, i.e., *L* = *Nλ*_0_/(*ε*_2_)^1/2^ (*N* can be any arbitrary positive integer, and *λ*_0_ denotes the wavelength in free space at the operating frequency *f*_0_). This is the FP resonance condition, which directly shows the TIM-like performance, i.e., guiding incident waves along the optic axis without introducing any reflection or transmission phase delay. It should be noted that such a TIM-like performance can always be observed at FP resonance frequencies, such as 2*f*_0_, 3*f*_0_, etc.

As an example, by setting the background to be the Teflon (*ε*_1_ = 2.01, *μ*_1_ = 1), *ε*_2_ = 1, *μ*_2_ = 1, *N* = 1, we show the calculated transmission amplitudes (solid lines) and phases (dotted lines) in Figure S1b at the frequencies *f*_0_ (blue lines) and 2*f*_0_ (red lines). Meanwhile, we also show the simulated magnetic field distributions in Figure S1c while placing a point source near this FP slab at *f*_0_ (I) and 2*f*_0_ (II). As we expect, both the calculated and simulated results show robust TIM-like performance at two FP resonance frequencies.

**
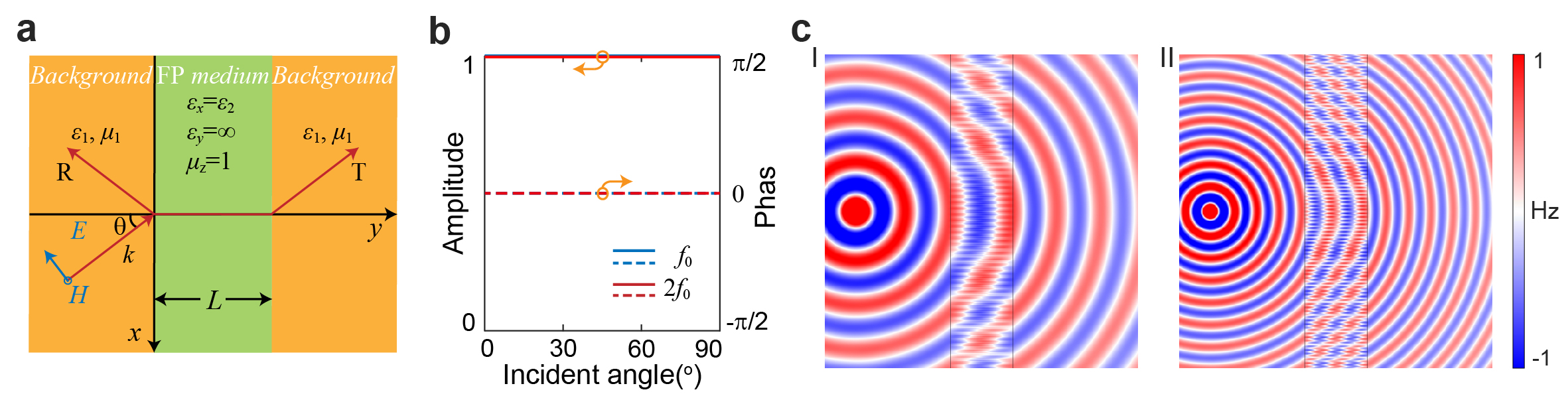
**

**Figure S1.** a) Schematic of an FP medium slab (*ε_x_* = *ε*_2_, *ε_y_* = ∞, *μ_z_*= 1) placed in a background (*ε* = *ε*_1_, *μ* = *μ*_1_). b) Transmission amplitudes (solid lines) and phases (dotted lines) of the FP slab at two FP resonance frequencies (blue lines for *f*_0_, red lines for 2*f*_0_). c) The magnetic field distributions at *f*_0_ (I) and 2*f*_0_ (II) while placing a point source near the FP slab.

**II. Inhomogeneous FP medium in the cylinder coordinate**

In this section, we analyze the impedance matching performance of the inhomogeneous FP medium in the cylinder coordinate while connecting two different dielectrics. To simplify the numerical calculation, we evaluate the scattering of an annulus inhomogeneous FP medium while enclosing a specific dielectric cylinder in free space.

As shown in **Figure S2**a, the inner and outer radii of the annulus FP medium are denoted as *ρ*_1_ and *ρ*_2_, respectively. Keeping the same as those shown in Equation (9) and (11) in the main text, the inner dielectric cylinder has the relative permittivity of (*ρ*_2_/*ρ*_1_)^2^, and the involved parameters of the FP medium under the TM wave incidence (with a *z*-polarized magnetic field) are:

, (S3)

where *N*_1_ can be any arbitrary nonzero integer or half-integer, and *λ*_0_ denotes the wavelength in free space at the operating frequency *f*_0_. Based on the Mie solution of the multiple concentric cylindrical layers,^[1]^ the total scattering width (normalized by 2*ρ*_2_) of such a rotationally symmetric structure can be easily numerically calculated. By choosing *ρ*_1_ = 0.721*λ*_0_, *ρ*_2_ = 1.442*λ*_0_, we calculate the total scattering widths of this composite cylinder as the function of the frequency with *N*_1_ = 1 (blue line) and *N*_1_ = 2 (red line), as shown in panel II of Figure S2a. With these parameters, *ε_φ_* varies from 4 to 1 when *N*_1_ = 1 and from 16 to 4 when *N*_1_ = 2, according to Equation (S3). As we see, extremely small scattering widths appear at the FP resonance frequencies, nearly independent of *N*_1_ at the FP resonance frequencies. However, the larger the value of *N*_1_, the smaller the fundamental FP resonance frequency. Previous results imply that we can choose a proper value of *N*_1_ in the actual design to ease the physical implementation without involving plasmonic media, since the impedances at the boundaries (*ρ* = *ρ*_1_ or *ρ* = *ρ*_2_) do not need to match. It should also be noted that there are also dips at *f*_0_/2, 3*f*_0_/2, etc., when *N*_1_ = 1. This is because the impinging waves always pass the FP medium twice.

For more details, we show the simulated magnetic field distributions at *f*_0_ and 2*f*_0_ when a plane wave is incident on this composite cylinder in Figures S2b and S2c, respectively. Panels I show the results with *N*_1_ = 1, and panels II show the results with *N*_1_ = 2. No observable scattering outside the cylinder can be seen in all cases, showing nearly perfect impedance matching.

**
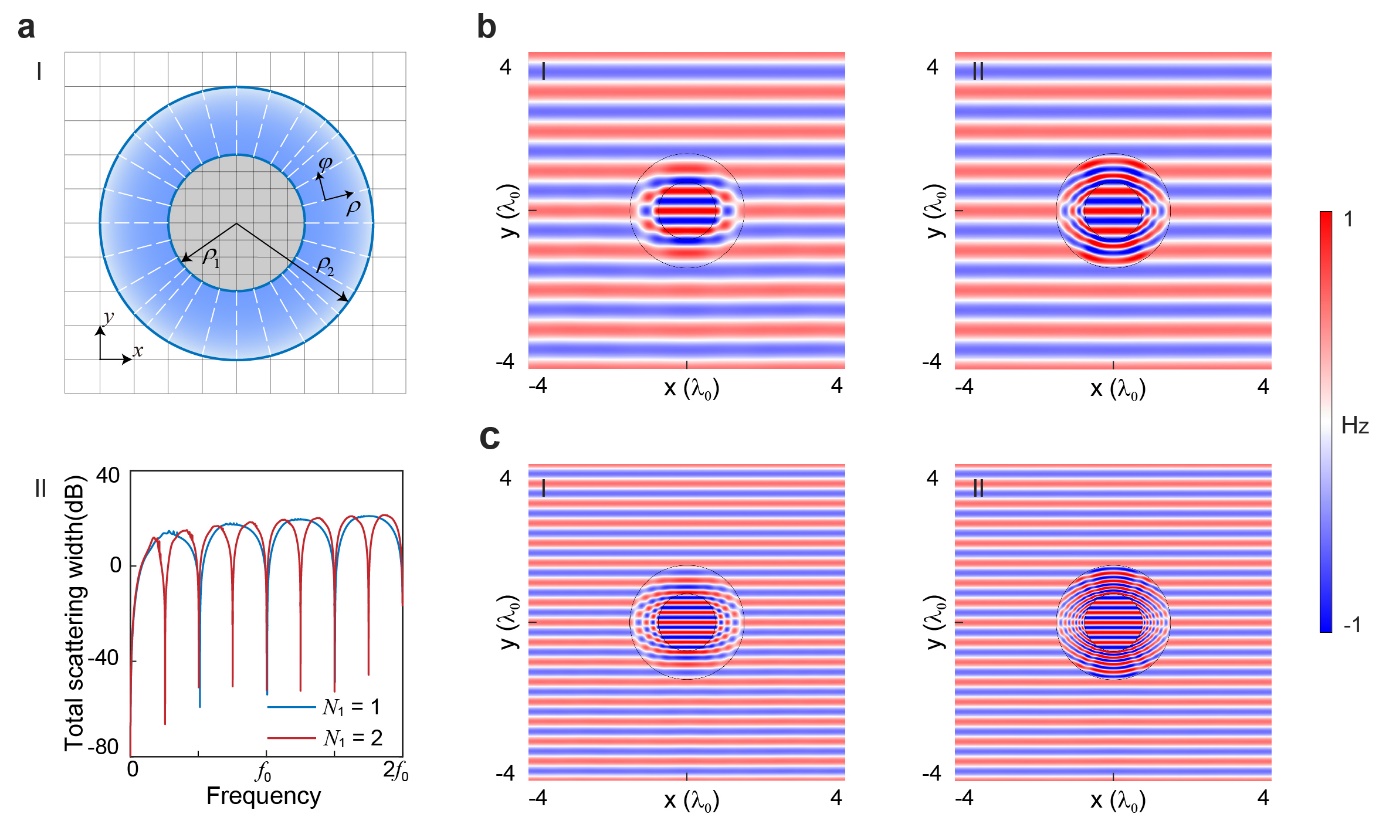
**

**Figure S2.** The performance of the annulus inhomogeneous FP medium in the cylinder coordinate. a) Schematic (I) and the numerically calculated total scattering widths (II) with different *N*_1_. b) Full-wave simulated magnetic field distributions with *N*_1_ = 1 (I) and *N*_1_ = 2 (II) at *f*_0_. c) The corresponding magnetic field distributions at 2*f*_0_.

**III. Derivation of the dielectric thickness in the annulus inhomogeneous FP structure**

In this section, we show the detailed derivation of the thickness function *d_ρ_* of the dielectric slab in the annulus inhomogeneous FP structure. As shown in Figure 3b in the main text, the *ρ*-dependent slot thickness *P_ρ_* = *ρ* × *θ_del_*, where *θ_del_* denotes the central angle in radians of each slot. Here, *θ_del_* = π/90 ≈ 0.0349, because the semicircular-annulus FP structure is divided into 90 slots in the actual design. Assuming the thickness (along the *ϕ* direction) of the filling high-permittivity dielectric slab (with a permittivity of *ε*_2_) in each slot is *d_ρ_*, according to the Maxwell Garnett mixing rule,^[2]^ the effective permittivity *ε_ϕ_* of this dielectric-air composite slab satisfies:

, with $m=\frac{d_{\rho}}{P_{\rho}-d_{\rho}}$ and *ε*_0_ = 1. (S4)

Substituting *P_ρ_* = *ρθ_del_* and Equation (9 and 11) in the main text into Equation (S4), we derive the *ρ*-dependent thickness function of the filling dielectric slab as:

$d_{\rho}=\varepsilon_{2}\rho\theta_{del}\left(\frac{1-\rho^{2}\ln^{2}\left(\rho_{2}/\rho_{1}\right)/\left(N_{1}\lambda_{0}\right)^{2}}{\varepsilon_{2}-1}\right)$. (S5)

In our actual design, *N*_1_ = 0.5, *ε*_2_ = 6.15, *ρ*_1_ = 21.58 mm, *ρ*_2_ = 42.53 mm, *λ*_0_ = 60 mm. Therefore, the thickness function *d_ρ_*  can be calculated as *d_ρ_* = 4.17×10^-2^*ρ* - 2.13×10^-5^*ρ*^3^.

**IV. The impacts of losses and frequency dispersions on the cloaking performance**

In this section, we quantify the impacts of the material losses and frequency dispersion of copper on the cloaking performance.

First, we simulate the transmittance and reflectance of the designed cloak by applying periodic boundary conditions on the left and right sides, while the involved materials are all lossless, i.e., the metals are modeled using the perfect electric conductor (PEC), and the loss tangents of dielectrics are 0. **Figure S3**a shows the simulation setup, and the port width is 14.5 cm (slightly larger than the cloak). Figure S3b shows the simulated transmittance (blue line) and reflectance (red line) with the incident angle *θ* = 0°, and the insets show the details around two optimal operating frequencies. It is seen that the operating frequencies appear at 5.1 GHz and 10.06 GHz, slightly deviating from the desired ones (5 and 10 GHz). Such a deviation may result from the fact that the periodicity of the metallic slot array in the FP structures are not small enough. Figure S3c shows the simulated magnetic field distributions at 5.1 GHz, 10.06 GHz, and 6.3 GHz (with a large transmittance) for comparison. We can see that no obvious wave distortion is observed at the two working frequencies (5.1 GHz or 10.06 GHz), and strong scattering occurs at 6.3 GHz.

Second, to quantify the impacts of the material losses and frequency dispersion of copper on the cloaking performance at two operating frequencies, we remodel the cloak by replacing PEC sheets with the real copper ones (the permittivity of copper is described by the Drude model: *ε*_r_ = 1 - *ω*_p_^2^/(*ω*^2^ + *iω*γ), with a plasma frequency *ω*_p_ = 1.34 × 10^16^ rad/s and a damping frequency γ = 1.45 × 10^14^ rad/s.^[3]^) and adding dielectric losses (F4B with a loss tangent of 0.0015, and Rogers *RO*4360G2^TM^ with a loss tangent of 0.0038, both of which are obtained in the datasheet). Panels I and II of Figure S3d show the simulated energy dissipations on the copper sheets (red lines) and dielectrics (green lines), and the total ones (blue lines), with *θ* = 0°. It is seen that the total energy dissipations are about 5.4% and 8.5% at the low and high frequencies, respectively. Moreover, we also show the simulated transmittances of the lossy cloak (dashed lines) and the lossless one (solid lines) at two optimal operating frequencies in Figure S3e, varying with the incident angle *θ*. Here, the incident angle *θ* only ranges from 0 to 120° due to the C3 symmetry of the designed cloak. The simulated results show that the lossless cloak maintains high transmittance (greater than 0.999) at two optimal operating frequencies for all incident angles, demonstrating the omnidirectional invisibility transparency. The transmittances of the lossy cloak are smaller but still larger than 0.91 within the whole angle range.


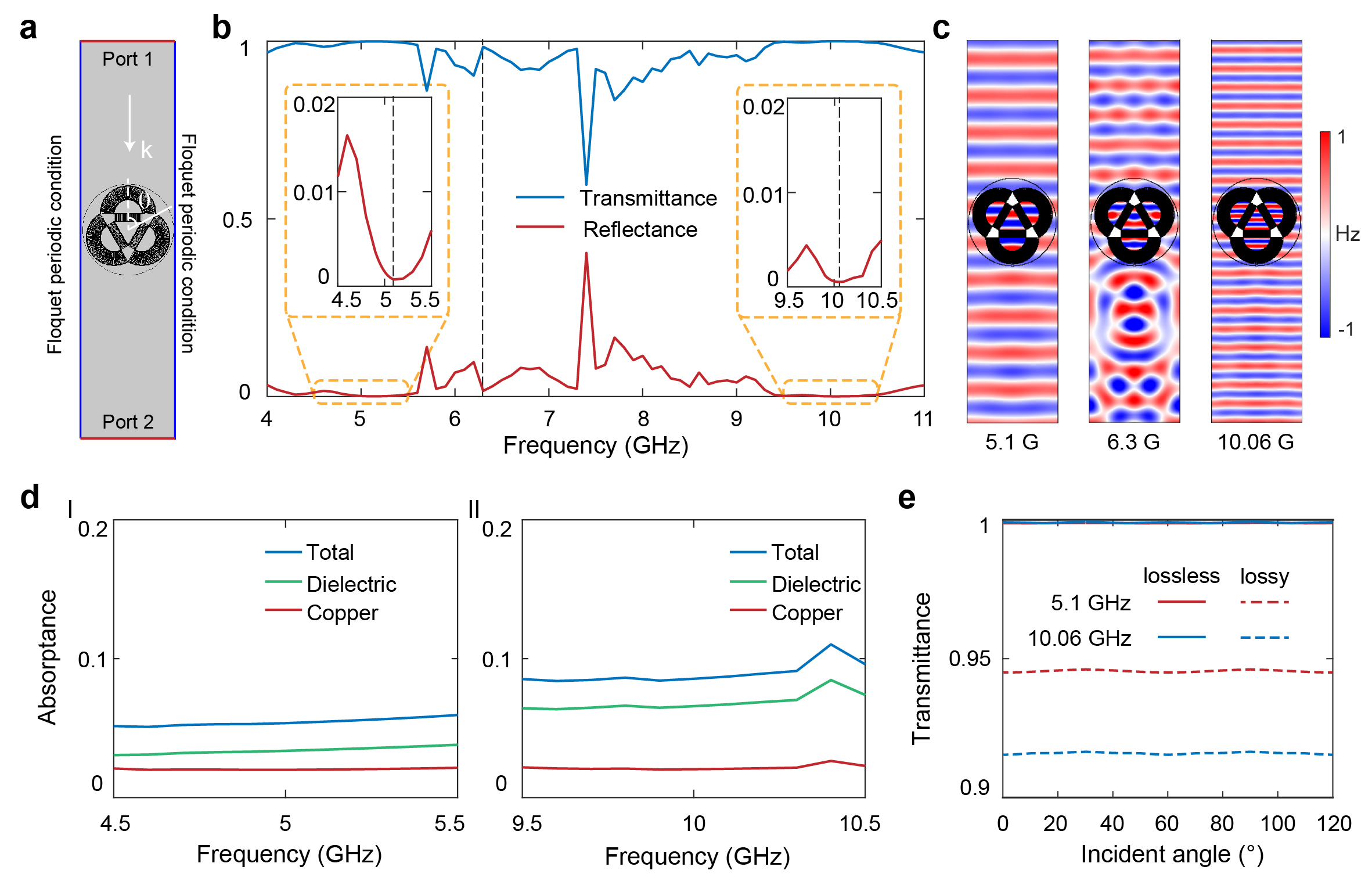


**Figure S3.** Quantitative analysis of the cloaking performance. a) Simulation setup while applying periodic boundary conditions. b) Simulated transmittance and reflectance varying with the frequency, with *θ* = 0°. c) Simulated magnetic field distributions at 5.1 GHz, 6.3 GHz and 10.06 GHz. d) Simulated energy dissipations on the dielectrics (green lines) and metals (red lines), and the total ones (blue lines) around 5 GHz (I) and 10 GHz (II). e) Simulated transmittances of the lossless cloak (solid lines) and the lossy one (dashed lines) varying with the incident angle *θ* at two optimal operating frequencies.

Finally, we also simulate the normalized total scattering width of the single cloak, as shown in **Figure S4**. The normalized total scattering width is calculated according to *σ_norm_*_._= , where *R* denotes the radius of the annulus FP structure, *H_s_* and *H_i_* denote the scattered and incident magnetic field intensities, respectively. Figure S4a shows the normalized total scattering widths of the lossless cloak (blue line), the lossy cloak (red line) and the bare object without cloak (green line), with respect to the frequency. It is seen that the scattering widths are greatly suppressed at 5.04 GHz and 9.98 GHz while covering the object with the lossless cloak or the lossy one, and the losses of dielectrics and the frequency dispersion of metals degrade the cloaking performance slightly (around 0.43 dB at 5.04 GHz, and 0.88 dB at 9.98 GHz). Figure S4b shows the normalized total scattering widths with respect to the incident angle at two optimal operating frequencies. Significant scattering suppressions are observed for all incident angles at the two optimal operating frequencies, and the impact of the losses and the frequency dispersion of metals is also small, showing a robust multiband cloaking performance.

*
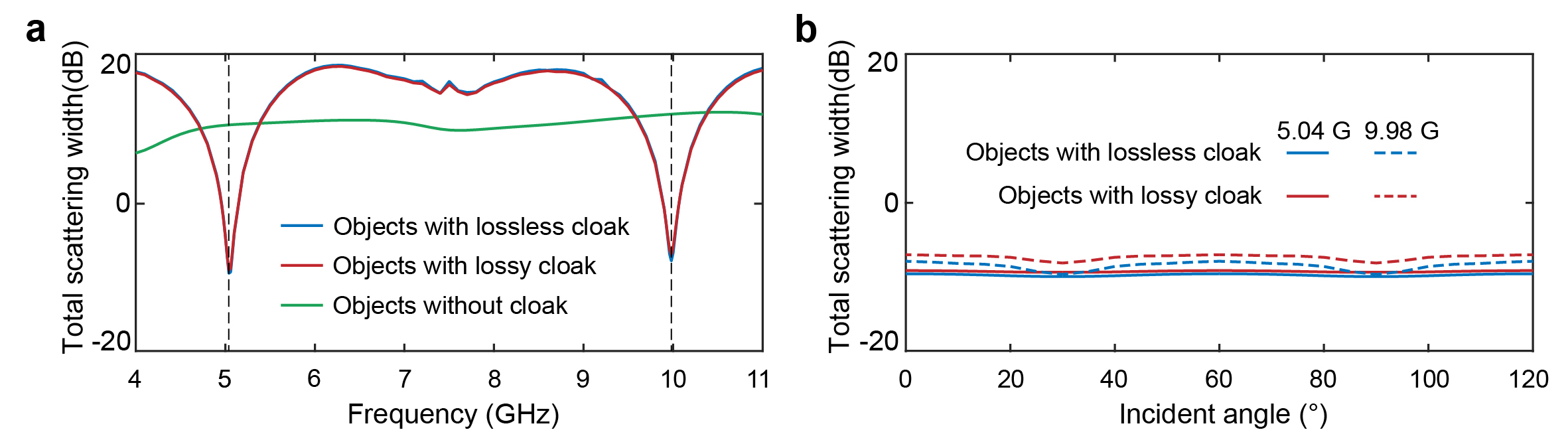
*

**Figure S4**. Simulated normalized total scattering widths of the single cloak. a) Normalized total scattering widths of the lossless cloak (blue line), the lossy cloak (red line) and the bare object without cloak (green line), with respect to the frequency. b) Normalized total scattering widths with respect to the incident angle at two optimal operating frequencies.

**V. Scattering magnetic field amplitude distributions around the designed sample**

**Figure S5** shows the simulated and measured scattering magnetic field amplitude distributions around the quadrangular metals with and without the cloak. Here, the scattering magnetic field amplitude is defined as *σ_with/without_* = |*H_with/without_*-*H_air_*|/|*H_air_*|, where *H_with_* and *H_without_* denote the total magnetic fields around the quadrangular metals with and without the cloak, respectively. *H_air_* denotes the magnetic field while removing all samples. The results in Figure S5 correspond to those shown in Figure 4 in the main text. We can see that the scatterings are obvious for the bare quadrangular metals but significantly suppressed in all simulations while covered with the designed cloak. The experimental results basically agree with the simulated ones but with degraded performances.


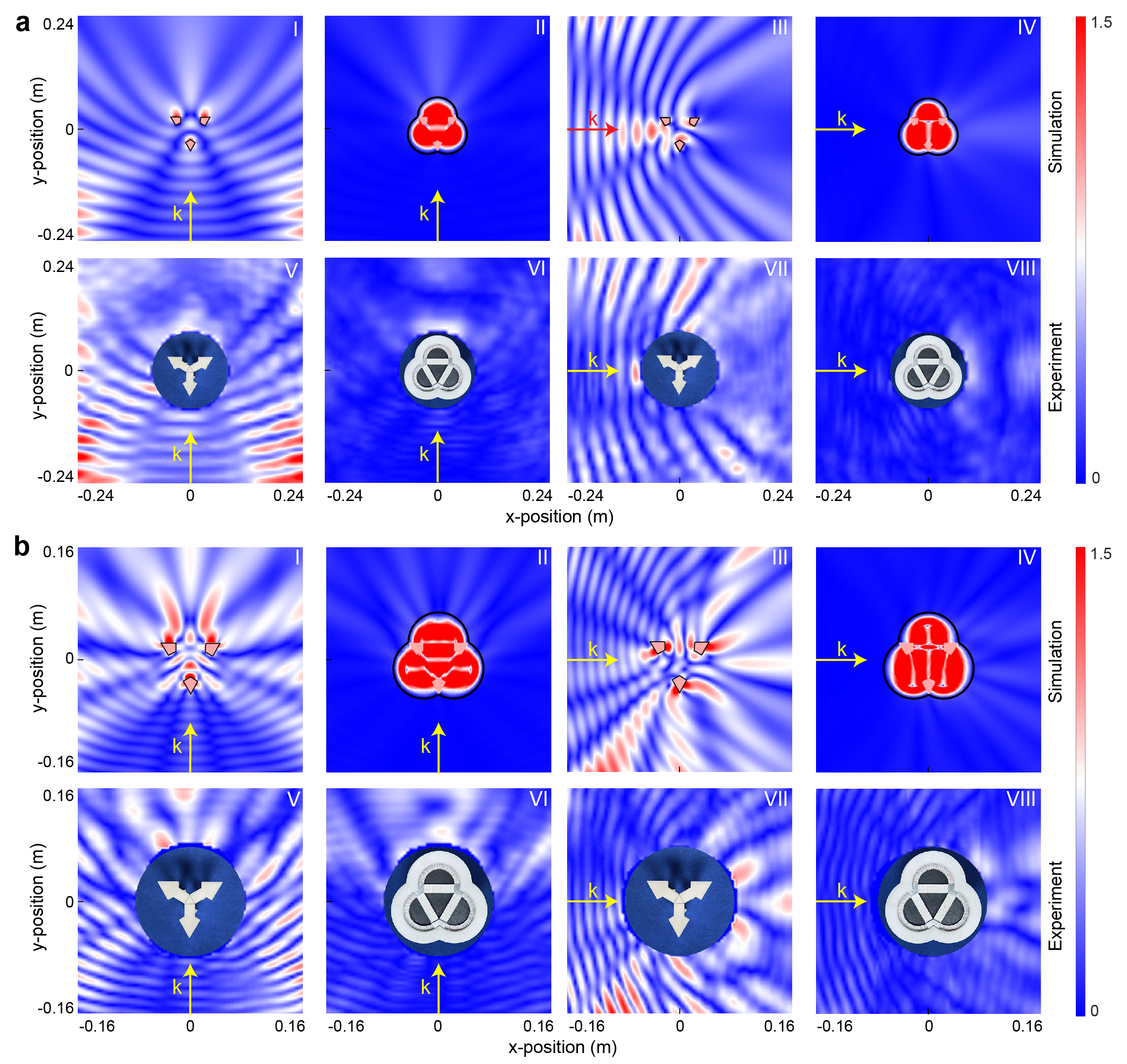


**Figure S5.** Simulated and measured scattering magnetic field amplitude distributions. a) Results at 5 GHz. Panels I-IV show the simulated scattering field distributions around three identical quadrangular metals without (I, III) and with the designed cloak (II, IV) at different incident angles. Panels V-VIII show the corresponding measured results. b) Corresponding results at 10 GHz.

**VI. Multiband cloak at terahertz frequencies**

In this section, we design a multiband omnidirectional cloak operating at terahertz frequencies to show the potential scaling of our strategy.

**Figure S6**a shows the schematic of the designed cloak comprised of silver sheets (described with the Drude model: *ε*_r_ = 1 - *ω*_p_^2^/(*ω*^2^ + *iω*γ), with *ω*_p_ = 1.39 × 10^16^ rad/s and γ = 3.12 × 10^13^ rad/s.^[3]^) and two dielectrics (silica and silicon), which operates at around 1 THz and 1.5 THz. Within this frequency band, the silica has a nearly non-dispersive permittivity (*ε*_1_ = 4, with a loss tangent of 1.5 × 10^-5^),^[4]^ and the silicon has a slightly dispersive permittivity (*ε*_2_ ranges from 11 to 11.3, with a loss tangent of 2.5 × 10^-3^).^[5]^ The pale blue regions (regions *A*′and *B*′) denote the silica, and the deep blue regions denote the silicon. The orange lines denote the silver sheets. Here, *ρ*_1_ = 156.6 μm, *ρ*_2_ = 306.6 μm, and the silver sheets are 0.1 μm in thickness. The planar FP structure (region *D*′) is constructed by closely stacking 54 silver-dielectric bilayers with a periodicity of 5.8 μm, and its details are schematically shown in the inset of Figure S6a. The annulus FP structure (region *C*′) is composed of 90 silver-dielectric bilayers with a periodicity of 5.47 μm at *ρ* = *ρ*_1_, whose details are schematically shown in the top panel of Figure S6b. The bottom panel of Figure S6b shows the thickness *d_ρ_* of the silicon slab dependent on *ρ*, with *d_ρ_* = 3.84 × 10^-2^*ρ* - 1.93×10^-7^*ρ*^3^. Such parameters ensure that all constitutive parameters satisfy Equation (8-12) in the main text with *N*_1_ = *N*_2_ = 1 at 1 THz.

Figure S6c shows the simulated magnetic field distributions at 1 THz (I) and 1.45 THz (II) when a point source with a *z*-polarized magnetic field is placed near the cloak. Although the shift of the higher operating frequency occurs due to the dispersive nature of silicon and silver, we still observe the remarkable multiband cloaking performance.

*
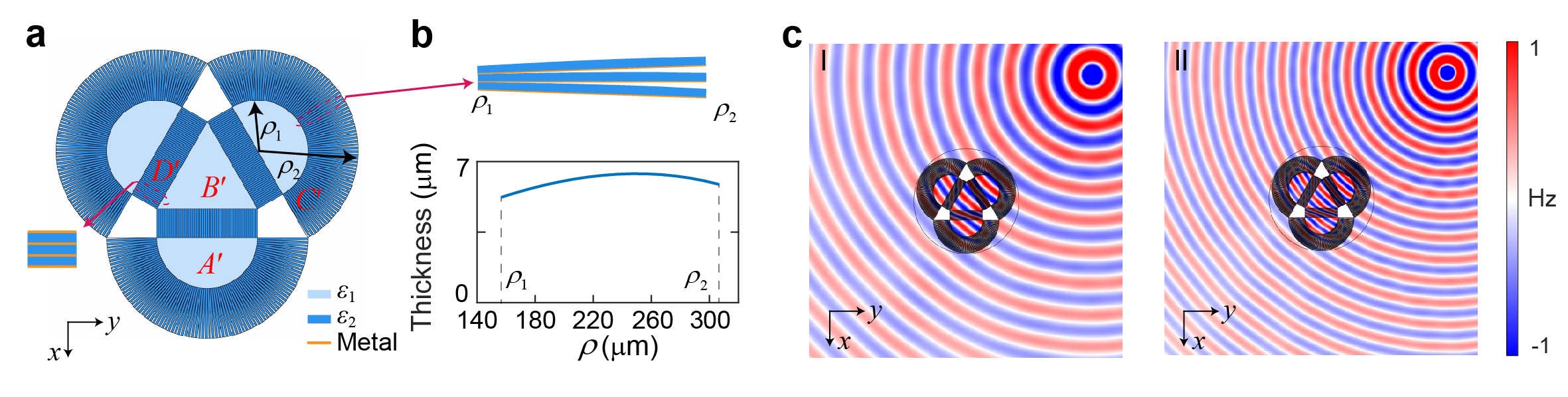
*

**Figure S6.** Multiband cloak operating at terahertz frequencies. a) The schematic of the cloak composed of silver sheets and two dielectrics (silica *ε*_1_ and silicon *ε*_2_). b) Details of the inhomogeneous annulus FP structure and the gradient thickness of the silicon slab varying with the radius *ρ*. c) Simulated magnetic field distributions while placing a point source near the cloak at 1 THz (I) and 1.45 THz (II).

**VII. Full-wave simulations**

All the simulated results shown in this work are performed using the commercial software COMSOL Multiphysics 5.6. In the simulation of Figure 1c, the relative permittivity along the optic axis of the TIM is set to 10000 rather than ∞, and the vertical permittivity and permeability are set to 0.0001 instead of 0. Similarly, in Figure 1e, the permittivity along the optic axis of the FP media is also set to 10000. In the full-wave simulations of the actual cloak shown in Figures 3c- 3e and Figure 4, all the copper sheets are set to be 0.035 mm in thick.

References

[1] C. A. Valagiannopoulos, P. Alitalo, *Phys. Rev. B* **2012**, *85*, 115402.

[2] A. Glisson, *IEEE Antenn. Propag. M.* **2000**, *42*, 72.

[3] M. A. Ordal, R. J. Bell, R. W. Alexander, L. L. Long, M. R. Querry, *Appl. Opt.* **1985**, *24*, 4493.

[4] R. Kitamura, L. Pilon, M. Jonasz, *Appl. Opt.* **2007**, *46*, 8118.

[5] M. Van Exter, D. Grischkowsky, *Appl. Phys. Lett.* **1990**, *56*, 1694.
